# The hand motor hotspot for seed-based functional connectivity of hand motor networks at rest

**DOI:** 10.3389/fnins.2022.896746

**Published:** 2022-08-12

**Authors:** Laura Bonzano, Marta Bortoletto, Agnese Zazio, Costanza Iester, Antonietta Stango, Roberto Gasparotti, Carlo Miniussi, Marco Bove

**Affiliations:** ^1^Department of Neuroscience, Rehabilitation, Ophthalmology, Genetics, Maternal and Child Health, University of Genoa, Genoa, Italy; ^2^Neurophysiology Laboratory, IRCCS Istituto Centro San Giovanni di Dio Fatebenefratelli, Brescia, Italy; ^3^Section of Neuroradiology, Department of Medical and Surgical Specialties, Radiological Sciences, and Public Health, University of Brescia, Brescia, Italy; ^4^Center for Mind/Brain Sciences - CIMeC, University of Trento, Rovereto, Italy; ^5^Section of Human Physiology, Department of Experimental Medicine, University of Genoa, Genoa, Italy; ^6^Ospedale Policlinico San Martino IRCCS, Genoa, Italy

**Keywords:** hand motor hotspot, hand motor networks, resting-state functional MRI, seed-based functional connectivity (FC), transcranial magnetic stimulation (TMS)

## Abstract

In the seed-based method for studying functional connectivity (FC), seed selection is relevant. Here, we propose a new methodological approach for resting-state FC analysis of hand motor networks using the individual hand motor hotspot (hMHS) as seed. Nineteen right-handed healthy volunteers underwent a transcranial magnetic stimulation (TMS) session and resting-state fMRI. For each subject, the hMHS in both hemispheres was identified by TMS with the contralateral abductor pollicis brevis muscle as the target, the site eliciting the highest and most reliable motor-evoked potentials. Seed regions were built on coordinates on the cortex corresponding to the individual left and right hMHSs. For comparison, the left and right Brodmann’s area 4 (BA4) masks extracted from a standard atlas were used as seed. The left and right hMHSs showed FC patterns at rest mainly including sensorimotor regions, with a bilateral connectivity only for the left hMHS. The statistical contrast BA4 > hMHS for both hemispheres showed different extension and lateralization of the functionally connected cortical regions. On the contrary, no voxels survived the opposite contrast (hMHS > BA4). This suggests that detection of individual hand motor seeds by TMS allows to identify functionally connected motor networks that are more specific with respect to those obtained starting from the *a priori* atlas-based identification of the primary motor cortex.

## Introduction

Functional connectivity (FC) is increasingly becoming a powerful means of identifying brain networks during the resting state. Resting-state functional magnetic resonance imaging (rs-fMRI) was first described by Biswal et al. (1995, 1997). As opposed to task-related fMRI, rs-fMRI does not require subjects to perform any specific task. The analysis of rs-fMRI data can be carried out through different approaches. Among these, the seed-based analysis consists of identifying a region of interest (ROI) called “seed” and then investigating its FC with all the other brain regions by computing the cross-correlation between the time-series of the seed and the rest of the brain. The disadvantage of this analysis is its dependence on selection of seeds, which makes it vulnerable to bias (Lv et al., 2018). The choice of the seed is usually made using either a functional method (functional seed) or an anatomical method (anatomical seed). In the former, functional seeds are identified by carrying out an active task. Indeed, for a study on resting motor networks, subjects are asked to perform a motor task during fMRI acquisition, and voxels resulting to be the most active (i.e., peaks of activation) and therefore related to brain areas mostly involved during the motor task are taken as seeds (Biswal et al., 1995; Xiong et al., 1999; Cordes et al., 2000; Jiang et al., 2004). However, it is worth noting that fMRI activity could be significantly influenced by task complexity (Haaland et al., 2004; Hausmann et al., 2004). In contrast, anatomical seeds can be identified using reference anatomical atlases, in Talairach or MNI space, such as the Anatomical Automatic Labeling template (Lowe et al., 1998; Zhang et al., 2009; Bonzano et al., 2015; Agarwal et al., 2016). In particular, to investigate hand motor networks, the “hand knob” area in the precentral gyrus, which is believed to be the primary hand motor region (Yousry et al., 1997), has been proposed as an appropriate seed. Anatomical and imaging studies locate this area in a region of the central sulcus (Puce et al., 1995; Yousry et al., 1997). To investigate possible differences in functional resting-state connectivity of the human motor network between right- and left-handers, Pool et al. (2015) used as seeds for the left and right primary motor cortexes (M1) the coordinates from the study of Hardwick et al. (2013). This study was based on an activation likelihood estimation meta-analysis of fMRI peak activations of seventy different motor learning experiments. Left and right M1s were identified on the rostral wall of the central sulcus at the hand knob formation.

Nevertheless, some studies showed that hand motor task-based activation is often localized outside this area (Hlustik et al., 2001; Siero et al., 2014; Ahdab et al., 2016; Hamidian et al., 2018). Furthermore, several studies opened a possibility that the hand knob may not be the optimum area for projecting maximum functional and structural connectivity between hemispheres (Hlustik et al., 2001; Siero et al., 2014). Hamidian et al. (2018) demonstrated that the maximal FC of the hand motor area between hemispheres occurs in the thumb area located laterally at the hand knob. Recently, a very elegant study investigated the FC, in healthy subjects, of two hand-knob sectors distinguished by different excitability, which was identified by high-frequency direct electrical stimulation delivered at rest on the hand-knob region in patients with tumor and undergoing intraoperative brain mapping (Simone et al., 2021). It is commonly accepted that hand motor hotspot (hMHS), investigated by transcranial magnetic stimulation (TMS), corresponds to the cortical motor representation of the hand (Barker et al., 1985; Di Lazzaro et al., 2004).

Nevertheless, it has been shown that the hMHS does not always correspond to the hand knob and anatomical M1 location (Ahdab et al., 2016; Siebner, 2020). Non-invasive brain stimulation studies showed that applying low-frequency repetitive TMS over the hMHS in the dominant motor cortex produced a more robust modulation effect on contralateral hemisphere activity and motor function than stimulation of the anatomical hand knob (Kim et al., 2020, 2021).

Following all these findings, here, we propose a new seed-based FC analysis of rs-fMRI data using individual seeds corresponding to the hMHS of each subject identified by TMS, with the contralateral *abductor pollicis brevis* (APB) muscle as the muscle target. Seed regions were built based on coordinates on the cortex corresponding to the individual left and right hMHSs. In addition, we considered an anatomical atlas and selected as reference two regions of interest (ROIs) corresponding to the left and right primary motor cortexes (Brodmann’s area 4), respectively.

Our aim was to demonstrate that detecting individual hand motor seeds by TMS allows to identify more specific functional motor networks with respect to those obtained starting from *a priori* atlas-based identification of the primary motor cortices.

## Materials and methods

### Participants

Twenty-one healthy right-handed volunteers were recruited and gave written informed consent to participate in the study as part of a larger study (Bortoletto et al., 2021). Inclusion criteria were right handedness, no history of neurological or psychiatric symptoms, and absence of contraindications to MRI and TMS. One participant did not undergo rs-fMRI acquisition, while another one was excluded from analyses because of technical problems during data acquisition. Therefore, nineteen participants were considered as the final sample of this study [mean ± SE; age = 33 ± 2 years; 8 F; Edinburgh Handedness Inventory (Oldfield, 1971) = 81.1 ± 3.6%]. Each subject underwent an MRI examination including rs-fMRI acquisition and a TMS session on 2 separate days within 2 weeks. This research was performed in compliance with the Declaration of Helsinki and was approved by the Ethical Committee of the IRCCS Istituto Centro San Giovanni di Dio Fatebenefratelli of Brescia and by the Ethical Committee of the Hospital of Brescia.

### Transcranial magnetic stimulation session

Each subject comfortably seated in a dimly lit room and underwent TMS for identification of hMHS in both the dominant and non-dominant brain hemispheres. The stimulation was performed with MagPro X100 including MagOption (MagVenture, Denmark) and set to deliver biphasic single pulses with a figure-of-eight C-B60 coil. The recharge delay was set at 500 ms. The coil was positioned tangentially to the scalp and with the handle pointing backward rotated away from the midline by approximately 45° so the current induced in the cortex followed the optimal direction, i.e., anterior to posterior and posterior to anterior (AP-PA).

The stimulation was assisted with a neuronavigation system (SofTaxic v.3.2; EMS, Italy), coregistering the T1 anatomical MRI to head position.

Electromyography of the APB muscles of the left and the right hands was visualized online by means of a bipolar belly tendon montage and a TMS-compatible system (BrainAmp; Brain Products GmbH, Munich, Germany). hMHS was functionally localized for each hemisphere as the position eliciting the highest and most reliable motor-evoked potentials (MEPs) in the contralateral APB as follows. First, the TMS coil was positioned over the hand knob area in the precentral gyrus, as identified on the individual MRI, and a few TMS pulses were delivered at 40% of the maximal stimulator output within an area of about 2 cm from the initial location. If no MEPs were induced, the TMS intensity was increased by 5%, and the procedure was repeated until MEPs of at least 50 μV were observed; at this point, the position of the coil was recorded in the neuronavigation system. Then, without varying the TMS intensity, a few TMS pulses were delivered moving the coil around the recorded position within an area of about 1 cm. The location inducing the highest and most reliable MEPs was finally recorded as hMHS. The entire procedure was performed for both hemispheres.

The neuronavigation system allowed to co-register the individual T1 anatomical MRI to head position. Then, once the coil was positioned on the scalp and the APB hotspot was identified, its exact location was recorded, and coil focus was projected perpendicularly to the coil plane on the gray matter surface of the individual MRI. This procedure provided by the neuronavigation system produced as output the corresponding coordinates both in the native space and in the MNI space.

### Magnetic resonance imaging session

Each subject underwent an MRI scan on a 3 T MR system (Skyra; Siemens, Erlangen, Germany). The MRI protocol included: axial T2-weighted fluid-attenuated inversion recovery [FLAIR; repetition time (TR) = 9,000 ms, echo time (TE) = 76 ms, inversion time = 2,500 ms, and voxel size = 0.6 mm × 0.6 mm × 4 mm] for assessment of possible incidental findings, and high-resolution T1-weighted 3D anatomical sequences (TR = 2,300 ms, TE = 2 ms, and 1-mm isotropic resolution).

For rs-fMRI, the subjects were told to stay still and not to perform any cognitive, language, or motor tasks while collecting axial T2*-weighted echo-planar imaging sequences sensitized for blood oxygen level-dependent (BOLD) contrast (TR = 1,000 ms, TE = 27 ms, 2.1-mm isotropic resolution, 600 volumes).

### Resting-state functional magnetic resonance imaging analysis

Preprocessing was performed using the Data Processing Assistant for Resting-State fMRI (DPARSF) (Yan and Zang, 2010), which is based on Statistical Parametric Mapping (SPM12^1^). The first 10 volumes of each subject were removed for signal equilibrium and adaptation of the participants to scanning noise; the remaining volumes were corrected for temporal differences and head motion (Friston 24-parameter model), and a mean functional image was obtained for each participant. No participant exhibited head motion greater than 1 mm maximum translation or 1° rotation throughout the course of scans.

Then, each subject’s T1-weighted structural image was co-registered to the mean functional image and was subsequently segmented. The obtained parameters were used to normalize functional images onto the Montreal Neurological Institute space.

After linear drift correction, a band-pass filter (0.01–0.1 Hz) was applied to reduce the effects of low- and high-frequency noises, followed by spatial smoothing with an isotropic Gaussian kernel of 4 mm full-width at half-maximum to decrease spatial noise. Six head motion parameters, white matter, and cerebrospinal signal were first regressed out to reduce the effects of head motion and non-neuronal BOLD signal fluctuations.

After the preprocessing of rs-fMRI data, seed-based FC analyses were performed using predefined seed regions.

In detail, we were interested in investigating resting-state functional connectivity (rsFC) motor networks in the dominant and non-dominant hemispheres, with specific attention to the hand motor representation. To this aim, separately for each subject, seed regions were built as spheres (6-mm radius) centered on the coordinates on the cortex corresponding to the individual left and right hMHSs (ROIs: l-hMHS and r-hMHS). The size of the seed regions was based on the TMS spatial resolution, which has been estimated within the range of 0.2–2 cm (Deng et al., 2013; Romero et al., 2019).

**FIGURE 1:**
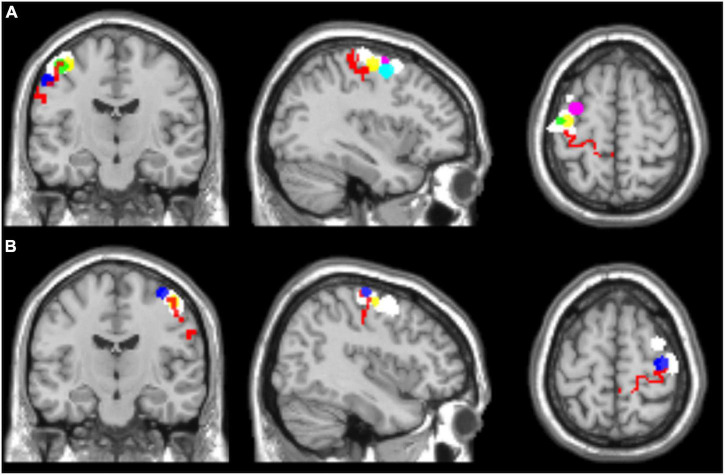
Regions of interest selected for seed-based functional connectivity analyses, overlaid on a T1 single-subject template. **(A)** Left hemisphere, **(B)** right hemisphere. l-BA4 and r-BA4 are shown in red; the other ROIs are differently colored to represent the individual seed regions based on the hand motor hotspots detected by TMS (l-hMHS and r-hMHS). Please note that some of the ROIs might be overlapped.

In addition, two ROIs including left and right Brodmann’s area 4, respectively, were selected for all the subjects from the WFU_PickAtlas (ROIs: l-BA4 and r-BA4). Figure 1 shows the selected ROIs for the left and right hemispheres.

For each participant, the averaged time course during the rs-fMRI period was extracted from the identified seed regions, and a correlation analysis was performed with every other voxel in the brain to assess the connectivity between these regions and the rest of the brain. The obtained correlation coefficient maps were z-score-transformed to improve the normality, generating a zrsFC map for each participant and each ROI (named ROI-zrsFC map).

### Statistical analysis

The resulting seed-to-whole-brain connectivity maps were introduced into a 2nd-level analysis to obtain the group maps. For each ROI, a one-sample *t*-test was performed by entering the ROI-zrsFC maps to detect brain areas displaying significant rsFC to the seed region at group level (*p* < 0.05 FWE-corrected, extent of threshold *k* = 20 voxels).

In order to compare the results obtained with the different ROIs separately for the two brain hemispheres, paired sample *t*-tests were conducted (l-hMHS vs. l-BA4 and r-hMHS vs. r-BA4; *p* < 0.05 FWE-corrected, extent of threshold *k* = 20 voxels).

## Results

Table 1 reports the gray matter location of the left and right hMHSs detected by TMS for each subject (MNI coordinates and corresponding BA). The points were used as centers of the spherical ROIs for the seed analysis specific for each subject. Brodmann’s areas included in each individual ROI are reported.

**TABLE 1:** Description of the ROIs obtained using the individual hand motor hotspot (hMHS): MNI coordinates and corresponding location of the hMHS detected by TMS for each subject in the left and right hemispheres and Brodmann’s areas within each ROI.

Laterality	Subject ID	hMHS MNI x y z (mm)	Location	Brodmann’s areas included in the ROI
Left	1	−54 −9 42	Gray matter	4, 3
	2	−43 −9 53	Gray matter	4, 6
	3	−31 1 57	Gray matter	4, 6
	4	−37 −9 54	Gray matter	4, 6
	5	−36 2 49	Gray matter	4, 6
	6	−46 −4 51	Gray matter	4, 6
	7	−45 −12 56	Gray matter	4, 6
	8	−51 −16 53	Gray matter	4, 3
	9	−43 −9 52	Gray matter	4, 6
	10	−43 −14 54	Gray matter	4, 3
	11	−39 −6 56	Gray matter	4, 6
	12	−43 −12 56	Gray matter	4, 6
	13	−39 −12 59	Gray matter	4, 6
	14	−49 −11 51	Gray matter	4, 3
	15	−41 12 50	Gray matter	4, 6
	16	−43 −12 54	Gray matter	4, 3
	17	−34 −17 62	Gray matter	4, 6
	18	−36 9 52	Gray matter	4, 6
	19	−22 −8 68	Gray matter	4, 6
Right	1	37 −14 60	Gray matter	4, 6
	2	54 −18 50	Gray matter	1, 3
	3	34 14 58	Gray matter	6
	4	46 −7 53	Gray matter	4, 6
	5	57 2 42	Gray matter	4, 6
	6	44 7 47	Gray matter	4, 6
	7	46 −17 59	Gray matter	4, 3
	8	49 −9 52	Gray matter	4, 6
	9	40 3 52	Gray matter	4, 6
	10	45 −17 55	Gray matter	4, 3
	11	34 1 59	Gray matter	4, 6
	12	44 −10 56	Gray matter	4, 6
	13	44 −14 58	Gray matter	4, 6
	14	48 −11 49	Gray matter	4, 3
	15	43 4 50	Gray matter	4, 6
	16	47 −5 53	Gray matter	4, 6
	17	41 −15 58	Gray matter	4, 6
	18	44 −3 50	Gray matter	4, 6
	19	49 5 43	Gray matter	4, 6

**FIGURE 2:**
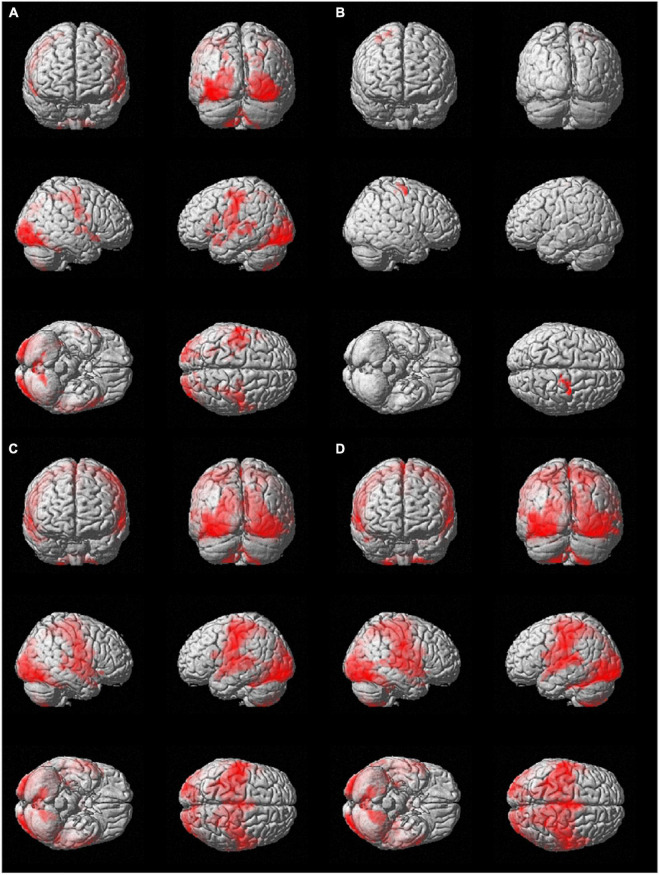
Group seed-to-whole-brain functional connectivity results using as seeds **(A)** the l-hMHS, **(B)** the r-hMHS, **(C)** the l-BA4, and **(D)** the r- BA4. Voxels significantly connected with the different seed regions are displayed on a rendered surface (*p* < 0.05 FWE-corrected, extent of threshold *k* = 20 voxels, height threshold *t* = 7.05).

The results from the seed-to-voxel second level analysis using the ROIs identified in the two hemispheres are displayed in Figure 2 (only suprathreshold voxels are shown, *p* < 0.05, FWE-corrected at the cluster level).

The use of the l-hMHS as seed produced clusters of correlated resting-state BOLD activity in both hemispheres. Specifically, the functional network included bilaterally the primary sensory and motor regions and the cerebellum (lobules VI, VIIb, and VIII, and Crus I). These networks also involved, in the left hemisphere, frontoparietal cortical regions (Figure 2A). The r-hMHS seed showed a FC pattern mainly related to ipsilateral sensorimotor regions (Figure 2B).

Significant clusters of FC during resting state with the left BA4 mainly included the left cerebellum (lobules VIIb and VIII), language processing areas (BA 38 and BA 20), amygdala, hippocampus, and putamen. A large cluster was found with a bilateral pattern covering the occipital, frontal (precentral gyrus), temporal (superior temporal gyrus), and parietal (postcentral gyrus) lobes, the insula, and the cerebellum (lobule VI, lobules IV-V, and Crus I) (Figure 2C).

When considering the right BA4 as the seed ROI, significant resting-state FC was found with the left amygdala and thalamus, and the right parahippocampal gyrus (BA 27). Similarly to what was found for the left BA4, a large cluster showed a bilateral pattern covering the occipital, frontal, temporal, and parietal lobes, and the cerebellum (lobule VIII, lobule VI, lobules IV-V, and Crus I) (Figure 2D).

**FIGURE 3:**
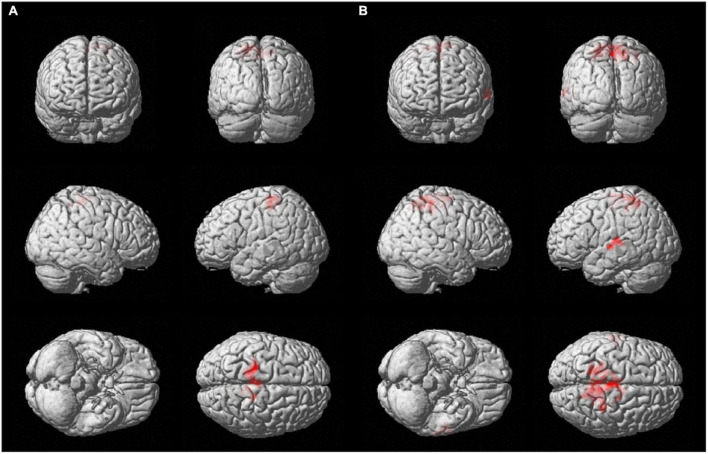
Surface rendering showing voxels surviving the statistical contrasts between the group seed-to-whole-brain functional connectivity maps obtained with the different ROIs separately for the two brain hemispheres (*p* < 0.05 FWE-corrected, extent of threshold *k* = 20 voxels, height threshold *t* = 7.05). **(A)** l-BA4 > l-hMHS and **(B)** r-BA4 > r-hMHS.

For both brain hemispheres, the statistical comparison of the rsFC of the seed ROIs located in the BA4 and the rsFC of the seed ROIs located in the hMHS revealed areas with significantly greater FC with the BA4. No voxels survived the opposite contrasts (l-hMHS > l-BA4 and r-hMHS > r-BA4). As shown in Table 2 and Figure 3, with respect to the left hMHS, the left BA4 was more functionally connected with the left primary motor cortex (BA4), bilateral primary somatosensory cortex (BA3) and supplementary motor areas (BA6), and the right associative cortex (BA5).

**TABLE 2:** Comparisons of the results obtained with the different ROIs as seeds, separately for the two brain hemispheres (l-hMHS vs. l-BA4 and r-hMHS vs. r-BA4; *p* < 0.05 FWE-corrected, extent of threshold *k* = 20 voxels).

Seed region	Cluster size (voxels)	*Z*-score	Peak MNI x y z (mm)	Laterality	Brodmann’s area
l-BA4 > l-hMHS	204	5.99	−15 −36 66	Left	4
		5.95	−21 −30 63	Left	3
		5.91	−6 −33 54	Left	6
	55	5.55	3 −39 54	Right	5
		5.55	12 −33 60	Right	6
		5.53	21 −30 60	Right	3
l-hMHS > l-BA4	*No voxels survived*				
r-BA4 > r-hMHS	563	5.93	6 −48 66	Right	5
		5.9	9 −39 69	Right	4
		5.73	−18 −51 51	Left	7
	63	5.7	−66 −27 3	Left	22
		5.49	−60 −12 0	Left	22
		5.32	−63 −21 9	Left	42
r-hMHS > r-BA4	*No voxels survived*				

“Peak” MNI coordinates refer to voxels showing most significant statistical difference between the functional connectivity maps obtained by the two seed-based analyses (with hMHS or BA4 as seed), cluster by cluster.

With respect to the r-hMHS, the r-BA4 was more functionally connected with the right associative (BA5) and primary motor (BA4) cortices, and the left associative cortex (BA7) and auditory and language processing areas (BA22 and BA42).

## Discussion

In this study, we proposed a new seed-based analysis on rs-fMRI data using individual seeds corresponding to the hMHS of each subject. TMS-induced hMHS was identified on the contralateral thumb muscle (i.e., APB muscle), demonstrated to have the maximal FC of the hand motor area (Hamidian et al., 2018). As shown here, there is variability among the subjects in line with previous studies (Niskanen et al., 2010; Ahdab et al., 2016). We think this further supports the usefulness of the proposed methodology for a more specific approach where the seed is identified by TMS as the “real” hMHS in a resting condition (overcoming the classic anatomical description of the human motor cortical representation that can be reproduced in standard atlases). Then, seed regions were built based on the coordinates on the cortex corresponding to the individual left and right hMHSs (named l-hMHS and r-hMHS, respectively).

Over the last decade, the use of neuronavigation combined with participants’ individual MRI has dramatically increased compared to landmark-guided navigation, leading to higher experimental control and reduced MEP variability (Julkunen et al., 2009; Sondergaard et al., 2021).

The approach of using the l-hMHS as a seed region revealed a bi-hemispheric motor network comprising sensorimotor areas as well as parts of the somatosensory and parietal cortexes, in line with Pool et al. (2015). From our analysis, we also found significant FC between the l-hMHS and the left and right cerebella as previously observed by Bonzano et al. (2015).

Using the l-BA4 as the seed region, we found, as expected, a FC map larger than what was found with the l-hMHS. Nevertheless, the l-hMHS showed FC patterns similar to the l-BA4. With respect to the l-hMHS, the l-BA4 was more functionally connected with ipsi- and contralateral sensorimotor areas. The contrast analysis l-BA4 > l-hMHS revealed that statistical differences were in intrahemispheric and interhemispheric connectivity strictly limited to sensorimotor and associative areas. This could be explained by the larger size of the l-BA4 seed region with respect to the individual l-hHMS. It is worth noting that the l-BA4 referred to the corticomotor representation of the whole right side of the body, and that the l-hHMS was specific for the right hand. Furthermore, the lack of voxels surviving the contrasts l-hMHS > l-BA4 suggests that the identification of individual hand motor seeds results in functionally connected motor networks that are more specific with respect to those obtained starting from the *a priori* atlas-based identification of the primary motor cortex.

The analysis of the rsFC of the r-hMHS seed region showed ipsilateral FC with sensorimotor areas, in particular premotor and supplementary motor areas. In contrast to what was found by Pool et al. (2015), the FC of the r-hMHS was not similar to what was observed for the l-hMHS seed region. Nevertheless, this is not surprising; in fact, the network asymmetry of hand motor areas has been previously demonstrated by rs-fMRI (Yan et al., 2012). Furthermore, the resting state FC analysis of the r-hMHS showed a difference in the extension and lateralization of the significantly functionally connected cortical regions with respect to the maps obtained for the r-BA4. The statistical comparison of the FC of the seed ROIs located in the r-BA4 and in the r-hMHS revealed areas with significantly higher FC with the r-BA4. Specifically, from the contrast analysis r-BA4 > r-hMHS, the r-BA4 resulted to be more functionally connected with left and right associative regions and the right primary motor cortex, and auditory and language processing areas than the r-hMHS. Indeed, it has been shown that pre-supplementary motor areas show resting-state to both cortical and sub-cortical language regions (Lou et al., 2017). This could suggest that the large seed region identifying the BA4 from atlas may include areas that are immediately close to the primary motor cortex making larger clusters of significant FC. As occurred for the contrast analysis in the left hemisphere, no voxels survived the opposite contrast (r-hMHS > r-BA4). This indicates that the r-hMHS was a subset of the r-BA4. In both cases, the FC maps resulting from the seed analysis based on the BA4 selected from an atlas are likely to include spurious connections, since the seed regions are very large and anatomically rather than functionally defined. On the other hand, the FC maps obtained with the hHMS seeds were a subset of what was obtained with the BA4 seeds. This underscored the meaningfulness of the results obtained with the innovative methodology proposed in our study and, at the same time, specificity (for the hand) and individuality (for the single subject).

Our findings demonstrate that using individual seed motor regions it is possible to investigate the FC of hand motor networks.

Moreover, we suggest that seed detection for resting-state analysis by TMS on the motor cortex in a resting condition is more specific than an anatomical region selected from an atlas and more realistic than using task-related fMRI data. In fact, the administration of TMS on the motor cortex can allow for the finding of the hand cortical motor representation in a resting-state condition (i.e., the subject is at rest and a stimulus of short duration is applied just for hundreds of microseconds). Thus, such seed detection is only related to the activity of the subject at rest.

Unfortunately, we did not acquire task-related fMRI here for a direct comparison with a different seed-based approach; however, we could suggest that a task-based approach can be more variable in the choice of the seed, because it is based on the peaks of significant clusters of activation, which can be influenced by task complexity.

In conclusion, resting-state FC analysis using the hMHS as seed can be a useful tool in the field of neuromodulation, where the identification of an individual motor network is crucial (Kim et al., 2020, 2021).

## Data availability statement

The raw data supporting the conclusions of this article will be made available by the authors, without undue reservation.

## Ethics statement

The studies involving human participants were reviewed and approved by the Ethical Committee of the IRCCS Istituto Centro San Giovanni di Dio Fatebenefratelli of Brescia and by the Ethical Committee of the Hospital of Brescia. The patients/participants provided their written informed consent to participate in this study.

## Author contributions

LB: conceptualization, investigation, formal analysis, writing – original draft, and supervision. MBr: conceptualization, investigation, formal analysis, and writing – original draft. AZ: investigation, formal analysis, visualization, and writing – review and editing. CI: formal analysis and writing – review and editing. AS: investigation and formal analysis. RG: investigation and writing – review and editing. CM: conceptualization, resources, and writing – review and editing. MBv: conceptualization, writing – original draft, and supervision. All authors contributed to the article and approved the submitted version.

## Footnotes

1
http://www.fil.ion.ucl.ac.uk/spm

## Abbreviations

APB: abductor pollicis brevis
AP-PA: anterior to posterior and posterior to anterior
BA: Brodmann’s area
BOLD: blood oxygen level-dependent
DPARSF: Data Processing Assistant for Resting-State fMRI
FC: functional connectivity
hMHS: hand motor hotspot
l-BA 4: left BA4
l-hMHS: left hMHS
MEPs: motor-evoked potentials
MNI: Montreal Neurological Institute
r-BA 4: right BA4
r-hMHS: right hMHS
ROI: region of interest
rsFC: resting-state functional connectivity
rs-fMRI: resting-state functional magnetic resonance imaging
TMS: transcranial magnetic stimulation.
